# Combined Aerobic and Resistance Exercises Evokes Longer Reductions on Ambulatory Blood Pressure in Resistant Hypertension: A Randomized Crossover Trial

**DOI:** 10.1155/2020/8157858

**Published:** 2020-07-20

**Authors:** Nayara Fraccari Pires, Helio José Coelho-Júnior, Bruno Bavaresco Gambassi, Ana Paula Cabral de Faria, Alessandra Mileni Versuti Ritter, Catarina de Andrade Barboza, Silvia Elaine Ferreira-Melo, Bruno Rodrigues, Heitor Moreno Júnior

**Affiliations:** ^1^Laboratory of Cardiovascular Pharmacology, School of Medical Sciences, University of Campinas (UNICAMP), Campinas, Brazil; ^2^Laboratory of Cardiovascular Investigation and Exercise, School of Physical Education (FEF), University of Campinas (UNICAMP), Campinas, Brazil; ^3^Departament of Internal Medicine and Geriatrics, Catholic University of Sacred Heart, Rome, Italy; ^4^Department of Physical Education, Ceuma University, São Luís, Brazil

## Abstract

**Aim:**

The present study compared the acute effects of aerobic (AER), resistance (RES), and combined (COM) exercises on blood pressure (BP) levels in people with resistant hypertension (RH) and nonresistant hypertension (NON-RH).

**Methods:**

Twenty patients (10 RH and 10 NON-RH) were recruited and randomly performed three exercise sessions and a control session. Ambulatory BP was monitored over 24 hours after each experimental session.

**Results:**

Significant reductions on ambulatory BP were found in people with RH after AER, RES, and COM sessions. Notably, ambulatory BP was reduced during awake-time and night-time periods after COM. On the other hand, the effects of AER were more prominent during awake periods, while RES caused greater reductions during the night-time period. In NON-RH, only RES acutely reduced systolic BP, while diastolic BP was reduced after all exercise sessions. However, the longest postexercise ambulatory hypotension was observed after AER (~11 h) in comparison to RES (~8 h) and COM (~4 h) exercises.

**Conclusion:**

Findings of the present study indicate that AER, RES, and COM exercises elicit systolic and diastolic postexercise ambulatory hypotension in RH patients. Notably, longer hypotension periods were observed after COM exercise. In addition, NON-RH and RH people showed different changes on BP after exercise sessions, suggesting that postexercise hypotension is influenced by the pathophysiological bases of hypertension.

## 1. Introduction

Hypertension refers to a highly prevalent multifactorial condition characterized by chronic elevations in blood pressure (BP) that is strongly associated with negative health-related outcomes [1, 2]. When hypertension occurs in the absence of other diseases (e.g., obesity) and without apparent cause, it is clinically denominated as primary hypertension (NON-RH) [2]. In this case, lifestyle changes and pharmacological therapy are commonly effective to blood pressure control [2, 3].

However, approximately 20% of the patients clinically diagnosed with NON-RH may present an inability to achieve BP levels lower than proposed cut-offs, regardless of the optimal dose of 3 or more antihypertensive drugs, including 1 diuretic, a condition denominated as resistant hypertension (RH) [4–6]. In the recent years, an increasing attention to RH has been paid by health professionals, given that these patients are at higher risk of cardiovascular events and death in comparison to people with NON-RH [4–6].

Hence, experts in the field [7] have suggested that complementary therapies may be important supporting actors in the management of RH patients, although no specific guidelines have been proposed. Physical exercise is a well-established strategy to control BP in NON-RH patients [8–15] and recognized as part of the hypertension therapy according to different medical associations [16, 17]. Indeed, findings from randomized clinical trials reported reduced BP levels in middle-aged and older adults after different exercise training protocols [8–15].

Notably, the beneficial effects of physical exercise are not restricted to its chronic practice since reduced BP levels may be observed after an acute session of exercise, a phenomenon denominated postexercise hypotension (PEH) [10, 13, 18]. PEH is found after aerobic (AER) and resistance (RES) exercises in NON-RH and normotensive people [19–22]. Furthermore, PEH may predict the success of exercise protocols [21, 22] and likely contribute to low cardiovascular risk during the performance of activities of daily living [23, 24].

The effects of physical exercise in RH are still poorly explored. A previous seminal study found postexercise ambulatory hypotension after AER exercise in patients with RH [25]. However, the effects of other regimes of exercise, such as RES, still seem to be elucidated. In addition, although no further cardiovascular benefits were observed in normotensive and hypertensive people after exercise sessions that combined (COM) AER plus RES exercises [26–28], this kind of exercise is recommended by experts in the field for people who aim to improve cardiorespiratory, musculoskeletal, and neuromotor fitness [29] and for hypertensive patients who intend to reach optimum blood pressure values [30].

Based on these premises, the present study investigated the acute effects of AER, RES, and COM on 24-hour ambulatory BP monitoring (ABPM) in people with RH and NON-RH. In addition, postexercise ABPM were compared between RH and NON-RH. Our hypothesis is that AER, RES, and COM might cause different patterns of PEH in RH and NON-RH.

## 2. Methods

### 2.1. Sample and Recruitment

This was a randomized controlled crossover trial that investigated the acute effects of AER, RES, and COM exercises on 24-hour ABPM in people with RH and NON-RH. Concealed randomized allocation into one of four experimental sessions was performed by an independent researcher using a simple computer-generated list of random numbers and an allocation ratio of 1 : 1 : 1 : 1. All researchers, including evaluators, exercise supervisors, and those responsible for statistical analysis, knew where participants were allocated. Participants were also aware of exercise session but were blind to study hypothesis.

Recruitment was carried out through the Outpatient Resistant Hypertension Clinic of University of Campinas (Campinas, SP, Brazil). Twenty patients agreed to participate in the study protocol: 10 subjects with stage 2 hypertension (NON-RH) and 10 subjects clinically diagnosed with RH. RH was defined as an uncontrolled BP despite the use of ≥3 antihypertensive medications at optimal doses, including a diuretic if possible, or patients with controlled BP using ≥4 antihypertensive medications [4–6]. The diagnosis of RH was assessed following a 6-month protocol for screening of secondary causes of hypertension (primary hyperaldosteronism, renal artery stenosis, pheochromocytoma, and obstructive sleep apnea) and pseudo-RH (counting pills and ABPM). The subjects carrying one or both conditions were properly excluded from the study. Patients who showed significant changes on electrocardiogram trace under resting or physical stress test; who dropped out from the investigation; who presented changes on antihypertensive medication in the past 6 months prior to inclusion in the study; who had cardiac or cerebrovascular diseases, heart failure, or renal dysfunction; who are practicing regular physical exercise over the 6 months preceding the beginning of the study; who are using hormonal replacement therapy; and who are smokers were excluded. We included male and female aged 40 to 80 years old able to practice physical exercises.

Sample size was estimated using G∗Power version 3.1.9.2. on the basis of the magnitude of the mean differences in SBP levels [25] among the three sessions in two repeated measures. Considering an ES set at 0.45 [25], a power of 80%, and a level of significance set at 5%, the sample size was estimated to be 10 participants per group.

This study was approved by the Research Ethics Committee of the Faculty of Medical Sciences, University of Campinas (Campinas, Brazil) (Protocol 1638486; registered at ClinicalTrials.gov under ID number NCT02987452), and all patients that met the eligibility criteria gave their informed written consent before participation. The investigation was performed according to the Helsinki Declaration of 1975 (as revised in 1983).

### 2.2. Procedures

Experiments were performed in a quiet air-conditioned room (22-24°C) always in the mornings (07:00-12:00 am) in the Laboratory of Cardiovascular Pharmacology of the University of Campinas. Experiments were separated into two distinct phases. In the first phase, participants completed a familiarization period to familiarize them with the proper technique of the physical exercises utilized in the present study. Afterwards, the optimal exercise load to AER, RES, and COM was determined. The familiarization period took place in 4-6 alternate days. In the second phase, participants were requested to come five times to the laboratory after a 12 h overnight fast, including water, energetic beverages, and alcohol consumption, and without performing intense physical activity for 24 h.

In the first visit, a Bioimpedance Analyzer 450 (Biodynamics Corporation, Seattle, USA) was used for anthropometric measurements [31–33]. Bioimpedance assessed body mass index, fat-free mass, fat mass, basal metabolic rate, and total body water content. In the following visits, participants performed an acute session of exercise (i.e., AER, RES, or COM) or control (CONT) according to prior randomization at least 1 h after a standardized light breakfast (i.e., 40 g chocolate mini-cookie (Bauducco, São Paulo, Brazil; 132 kcal, 18 g of carbohydrate, and 2.1 g of protein; 5.7 g of fat), 200 mL chocolate box milk (Toddynho, PepsiCo, São Paulo, Brazil; 167 kcal, 27 g of carbohydrate, and 3.7 g of protein; 5.1 g of fat), and 144 g brown crackers pack (Club Social Nabisco, São Paulo, Brazil; 110 kcal, 16 g of carbohydrate, and 1.9 g of protein; 4.4 g of fat)). Approximately one hour after the end of the exercise session, participants were lying comfortably in the supine position, instrumented to ABPM, and were discharged.

### 2.3. Primary Outcome

#### 2.3.1. Ambulatory Blood Pressure Monitoring

ABPM was recorded for a 24-hour period using the Spacelabs equipment 90217 (Spacelabs Inc., Redmond, WA, USA). Awake-time was considered the interval between the first and tenth hours after the experimental sessions, while night-time referred to the period between the eleventh and eighteenth hours after the experimental sessions. Participants were instructed to maintain and record normal daily activities in a personal diary.

#### 2.3.2. Exercise Session Protocols

Exercise protocols were based on American College of Sports and Medicine (ACSM) guidelines [15, 29]. AER, RES, and COM exercises were used in the present study. Exercise protocols were equalized according to the total session time.

A minimum interval of 96 hours was required between exercise sessions. AER exercise was performed in a treadmill for 45 minutes at 50-60% of maximal heart rate (HRmax) obtained from the ergometric stress test. HR was monitored continuously across the exercise session using a cardiac monitor (Polar RS800 CX, Polar Electro Oy, Kempele, Finland). RES exercise consisted of 6 exercises with 4 sets of 12 submaximal repetitions performed at moderate intensity (3-5 on the adapted Borg scale) [34] (i.e., 1^st^ chair squat, 2^nd^ vertical bench press, 3^rd^ seated knee raise, 4^th^ seated row, 5^th^ dorsiflexion and plantar flexion, and 6^th^ shoulder abduction). A 1-minute interval was adopted between sets and exercises. All exercises were performed in the total range of motion and muscle contractions—concentric and eccentric—and were performed at moderate velocity (2 sec for each). Participants were instructed to avoid the Valsalva maneuver during the entire muscle contraction. COM exercise consisted of a session of AER exercise performed at 50-60% HRmax for 25 minutes plus a session of RES based on 6 exercises with 2 sets of 12 submaximal repetitions at moderate intensity according to the modified Borg scale [34]. An experienced exercise physiologist supervised all exercise sessions.

The optimal loads for exercise sessions were determined the familiarization period. RES was acquired using the rating of perceived exertion (RPE) method [35] based on the resistance of the elastic bands proposed by Uchida et al. [36]. A maximal exercise stress test on a treadmill using an individualized incremental protocol was used to determine the intensity of AER exercise. Before exercise testing, participants remained seated for 20 min. A resting electrocardiogram was performed, and BP, HR, and lactate levels were assessed. Afterwards, the incremental test using an electronic treadmill (Life Fitness®, model 9700HR®, Fort Mill, Tennessee, USA) was initiated, according to a modified Bruce protocol, which included six stages with 3 min each, characterized by increasing speed (2.7-6.8 km/h) and grade (0-16%). The HRmax were considered the highest HR recorded at the exhaustion moment. Electrocardiograph patterns were registered and accompanied by a cardiologist throughout the whole test.

#### 2.3.3. Control (CONT) Session

CONT session involved the continuous monitoring of blood pressure to over 60 minutes. This evaluation was considered the baseline values for all comparisons. Afterwards, the ABPM was removed, and the participants remained seated, but not exercising, in the machines for another 60 minutes.

### 2.4. Statistical Analysis

Normality of data was tested using the Shapiro-Wilk test. Baseline comparisons among groups were performed using unpaired Student's *t*-test. A two-way ANOVA followed by a Bonferroni post hoc test was performed to identify differences among the different times of evaluations in the experimental sessions. The area under the curve (AUC) was calculated. Peaks less than 10.0% of the distance from minimum to maximum *Y* were ignored. ANOVA followed by a Bonferroni post hoc test was performed to identify differences among experimental sessions. Categorical variables were presented in frequencies and/or percentages and compared by chi-squared test. All statistics analyses were performed using the GraphPad Prism 6.0 (GraphPad Prism Inc., 2000). All statistical methods are two-tailed, *P* values were calculated, and statistical significance was set at ≤0.05.

## 3. Results

Participant recruitment and experimental sessions were conducted from January 2017 to December 2017. No exclusions or dropouts occurred after randomization, and no patients reported changes in antihypertensive medication during the follow-up examination. Participants completed all experimental sessions.

Characteristics of study participants are shown in Table 1. No differences in clinical characteristics were observed among the groups. Higher glucose levels were observed in NON-RH, while people with RH had higher HDL-c levels. As expected, all patients with RH were under diuretic treatment. However, diuretic was only taken by 5 participants in the NON-RH group and it was lower in RH (*P* = 0.03). No other differences were found between the groups.

### 3.1. Effects of an Acute Session of Exercise on 24-Hour ABPM of RH Patients

**Figure 1:**
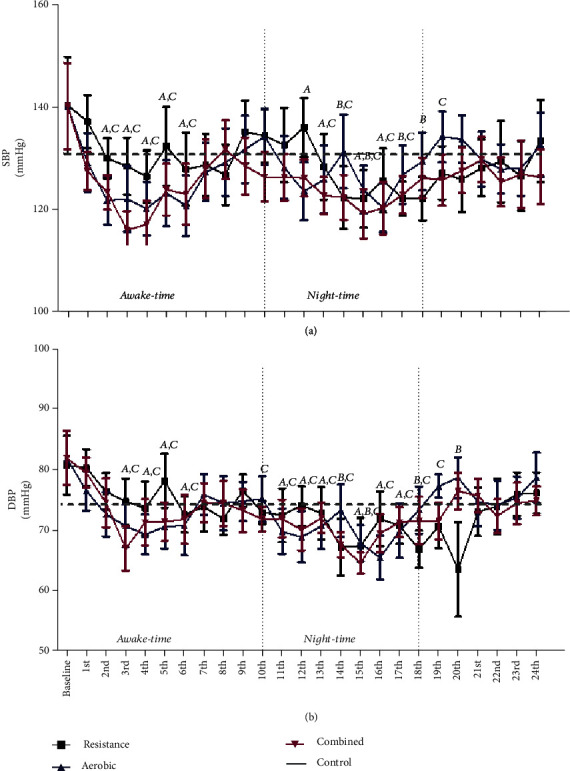
Ambulatory systolic blood pressure (a) and diastolic blood pressure (b) levels after aerobic, resistance, and combined exercises in RH. ^a^*P* < 0.05 aerobic in comparison to baseline values; ^b^*P* < 0.05 resistance in comparison to baseline values; ^c^*P* < 0.05 combined in comparison to baseline values.

Figure 1 shows 24-hour ABPM in RH patients. There were no significant within- and between-group differences in systolic (Figure 1(a)) and diastolic (Figure 1(b)) ABPM at baseline and after experimental sessions. Systolic BP was significantly reduced in all exercise groups when compared to baseline. AER reduced systolic BP from the 2^nd^ to 7^th^ (Δ = −17.6 mmHg), 12^th^ to 13^th^ (Δ = −15.4 mmHg), and 15^th^ to 16^th^ (Δ = −18.0 mmHg) hours after exercise. A shorter PEH period was observed after RES, given that significant reductions were only found from the 14^th^ to 15^th^ (Δ = −19.5 mmHg) and 17^th^ to 18^th^ (Δ = −15.3 mmHg) hours after exercise. On the other hand, the longest PEH period was observed after COM, so that significant reductions were observed from the 2^nd^ to 6^th^ (Δ = −19.5 mmHg), 13^th^ to 17^th^ (Δ = −18.6 mmHg), and 19^th^ (Δ = −14.3 mmHg) periods. Diastolic BP was also reduced after acute exercise protocols. PEH was observed after AER from the 4^th^ to 6^th^ (Δ = −11.6 mmHg), 11^th^ to 13^th^ (Δ = −12.0 mmHg), and 15^th^ to 17^th^ (Δ = −14.1 mmHg) hours after exercise. RES caused significant PEH from the 14^th^ to 15^th^ (Δ = −11.3 mmHg), 18^th^ (Δ = −8.5 mmHg), and 20^th^ (Δ = −3.2 mmHg) hours after exercise. As observed in systolic BP, COM caused the longest PEH period: from the 3^rd^ to 6^th^ (Δ = −11.5 mmHg) and 10^th^ to 19^th^ (Δ = −11.7 mmHg) hours after exercise. There were no significant differences in systolic and diastolic BPs after CONT.

### 3.2. Effects of an Acute Session of Exercise on 24-Hour ABPM of NON-RH Patients

**Figure 2:**
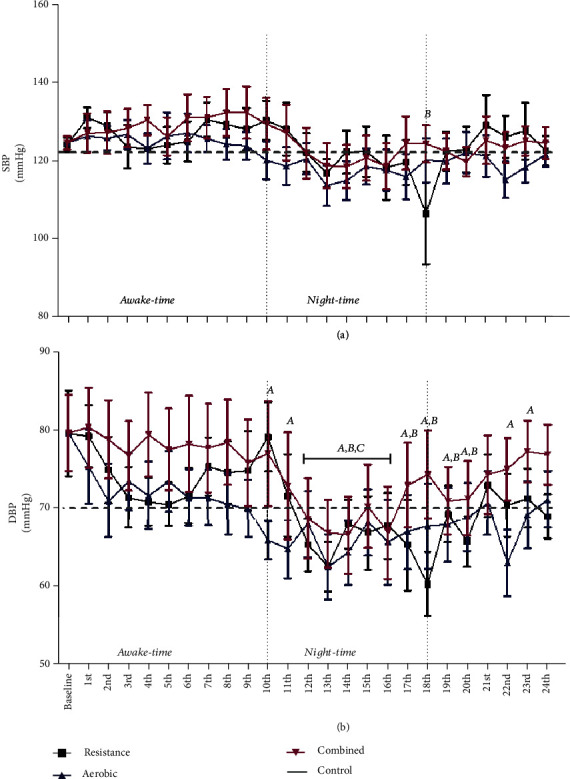
Ambulatory systolic blood pressure (a) and diastolic blood pressure (b) levels after aerobic, resistance, and combined exercises in NON-RH. ^a^*P* < 0.05 aerobic in comparison to baseline values; ^b^*P* < 0.05 resistance in comparison to baseline values; ^c^*P* < 0.05 combined in comparison to baseline values.

Figure 2 shows 24-hour ABPM in NON-RH patients. No significant differences in systolic and diastolic BPs were observed among the groups at baseline or after exercise. AER and COM did not cause significant reductions on systolic BP (Figure 2(a)). On the other hand, RES reduced systolic BP in the 18^th^ moment relative to baseline (Δ = −18.0 mmHg). Diastolic BP (Figure 2(b)) was reduced after the exercise protocols. AER caused significant PEH from the 10^th^ to 20^th^ (Δ = −13.3 mmHg) and 21^st^ to 22^nd^ (Δ = −12.9 mmHg) hours after exercise. A shorter PEH period was observed after RES: from the 12^th^ to 20^th^ (Δ = −12.9 mmHg) periods. COM reduced diastolic BP from the 12^th^ to 16^th^ (Δ = −11.8 mmHg) hours after exercise. There were no significant differences in systolic and diastolic pressures after CONT.

### 3.3. Area under the Curve

Table 2 shows the results of AUC. In RH, SBP had a significantly lower AUC after COM relative to AER and RES in the 24 h period, awake-time, and night-time. RES had a lower AUC of SBP during the night-time period in comparison to AER. The AUC of DBP was lower in COM relative to AER and RES in the 24 h and awake-time periods and lower in RES when compared to AER over 24 hours. On the other hand, lower AUC of DBP was observed after AER relative to RES during awake-time. Regarding NON-RH, the AUC of SBP and DBP was higher in COM compared to AER and RES in all periods. In addition, the AUC of SBP in the 24 h and awake-time periods, as well as the AUC of DBP in the awake-time period, was lower in AER relative to RES.

### 3.4. Comparisons between RH and NON-RH

There were no significant differences in systolic and diastolic BPs between RH and NON-RH after acute exercise sessions.

### 3.5. Harms

There were no harms or other unintended effects of the intervention.

## 4. Discussion

The main findings of the present study indicate that an acute session of AER, RES, and COM exercises significantly reduced ambulatory BP in RH patients. Notably, longer reductions in systolic and diastolic BPs were observed after COM (~12 h) relative to AER (~6 h) and RES (~3 h) exercises. According to AUC analysis, COM reduced SBP in both awake-time and night-time periods in comparison to AER and RES. AUC analysis also indicated that the effects of AER exercise were more predominant in the awake-time period, while lower DBP was observed after RES in the night-time period. People with NON-RH showed different BP responses to exercise sessions. Indeed, systolic BP was only significantly reduced after RES. Diastolic BP was reduced after all exercise sessions, but the longest reductions were observed after AER (~11 h) in comparison to RES (~8 h) and COM (~4 h). AUC indicated that the lower diastolic BP values after AER were mainly observed in the awake-time period. Although different BP responses were observed between RH and NON-RH, no significant differences were observed among groups.

Findings of the present study are partially supported by Santos et al. [25], who reported that an acute session of AER exercise (45 minutes at 50% HRmax) reduced systolic (Δ = −4.7 mmHg) and diastolic (Δ = −4.0 mmHg) BPs in RH patients. Particularly, a greater and longer reduction is observed in the present study relative to Santos et al. [25].

These different results do not seem to occur due to baseline BP levels or designs of AER exercise, given that participants of both studies showed similar baseline ABPM values (~145 mmHg) and performed AER exercise sessions with similar volume and intensity. Hence, a possible explanation for these distinct results may be the amount of exercising muscle mass [37, 38]. In fact, the current AER session was performed in a treadmill, while Santos et al. [25] used a cycle ergometer. Nevertheless, these assumptions are only speculative and should be further investigated in future studies.

This is the first study that investigated the acute effects of different types of exercise on ABPM. Our findings bring new light to the exercise cardiology field by demonstrating that COM exercise elicits longer reductions in systolic and diastolic BPs compared to AER and RES. COM reduced ABPM over 24 hours, including awake-time and night-time periods. On the other hand, the effects of AER exercise occurred predominantly in the awake-time period, while larger and longer blood pressure reductions were observed in the night-time period after RES. Taken as a whole, these findings suggest that COM exercise might provide further benefits relative to AER or RES exercise alone in RH patients.

Earlier studies [26–28] in normotensive people and patients with NON-RH reported different results. Ferrari et al. [28], for example, found greater PEH in the awake-time after AER exercise when compared to COM exercise. Similarly, NON-RH patients of the present study showed longer PEH after AER in comparison to RES and COM. These findings suggest that different mechanisms are underlying the effects of physical exercise in RH and NON-RH.

A possible explanation for the greater blood pressure reductions observed after AER exercise is based on the fact that this kind of exercise reduces cardiac output (CO) without changing peripheral vascular resistance (PVR) [27, 39, 40]. By contrast, PVR seems to remain elevated after RES and COM exercises, suggesting that RES may reduce the postaerobic exercise hypotension [27, 39, 40].

On the other hand, RES seems to potentiate postaerobic blood pressure reductions in RH. Only one study investigated the possible mechanisms underlying blood pressure changes after exercise in RH, and researchers [25] reported discrete changes in vascular responses after AER exercise. This phenomenon would imply reduced systolic volume (SV) due to a reduced preload and, consequently, decreased CO. In addition, a decreased vascular resistance certainly implies reduced PVR. In this context, it is possible to suggest that RES not necessarily causes additional reductions on CO and PVR but preserves their changes in response to AER for a long period.

Nevertheless, these inferences must be interpreted with caution because they were based on different populations and in mechanisms that were investigated in the first hour after exercise sessions. In addition, CO and PVR have demonstrated an important intersubject variability [40], which indicates that specific studies are necessary to investigate the effects of different regimes of exercise on hemodynamic parameters of RH patients.

The cellular mechanisms underlying blood pressure reduction after exercise sessions were not investigated in the present study. However, many of them may likely have influenced our findings. The role of the activation of H1 and H2 histamine receptors on blood pressure control during [41] and after [42–44] AER exercise has been described in the literature. According to experts in the field, blockade of both histamine H1 and H2 receptors may inhibit post-AER hypotension in normotensive people [42, 43] and contribute to greater diastolic blood pressure values after exercise in black people [44]. This phenomenon seems to occur due to the fact that histamine receptor blockers significantly reduce vascular conductance in response to physical stress [43, 44]. Contrarily, blockade of H1 and H2 receptors seems not to affect blood pressure responses to RES [43].

Acute improvements in cardiac autonomic modulation are another possible mechanism mediating exercise-induced blood pressure reduction, although studies have reported conflicting results. Liu et al. [21] reported that an acute session of AER caused rebalancing of the sympathovagal modulation to the heart in prehypertensive people, while Teixeira et al. [27] found increased sympathovagal balance after AER in normotensive people. On the other hand, no changes on cardiac autonomic modulation were observed by de Brito et al. [45] after AER performed both in the morning and in the evening in prehypertensive men.

On the other hand, studies have reported increased sympathetic activity and sympathovagal balance in the first hour after RES in untrained normotensive [19, 39, 46] and hypertensive [39] people, as well as RES-trained people with hypertension [47, 48]. Although this phenomenon might contribute to increase CO and inhibit blood pressure reduction, studies [19, 39, 46–48] reported significant PEH, which might occur due to a compensatory reflex vasodilation [48].

Only a few studies investigated the effects of COM on autonomic modulation. Teixeira et al. [27] and Saccomani et al. [49] found increased sympathovagal balance after COM in normotensive people. Notably, Teixeira et al. [27] reported that COM caused a greater and longer increase in sympathetic activity and reduction in vagal activity relative to AER and RES. In hypertensive people, COM did not change autonomic modulation [50].

Taken together, this information might suggest that histamine and cardiac autonomic modulation are mediating PEH after COM in RH, while reduction on blood pressure after RES in NON-RH is at least predominantly mediating by a compensatory reflex vasodilation. Nevertheless, generalizations are inappropriate and future studies are needed to confirm our premises.

A question that remains unanswered is why RES and AER exercises reduced BP in different periods of the day in patients with RH. The behavior of BP throughout the day after exercise performance is still under debate. According to Nishiguchi et al. [2], reduced ambulatory BPs after AER are commonly observed during waking periods. In contrast, the effects of RES are heterogeneous [2]. Tibana et al. [51] support the findings of the present study by reporting significant blood pressure reduction during the night-time period in older women with metabolic syndrome after an acute session of RES.

These findings might indicate that RES and AER exercises affect differently the circadian cycle and the release of possible mechanisms associated with blood pressure reduction (e.g., neurohumoral factors, autonomic modulation) [51], thus causing reductions in BP in different periods of the day in people with RH. However, these inferences should be further investigated and tested in future studies.

The divergent blood pressure responses to exercise sessions between RH and NON-RH may also occur due to antihypertensive medication, given that all patients with RH were under diuretic treatment, while this class of antihypertensive medication was only taken by five participants in the NON-RH group. Indeed, authors have proposed that acute exercise combined with antihypertensive medication may cause greater PEH than exercise alone.

Anunciação et al. [50] observed that an acute session of supramaximal interval AER exercise combined with angiotensin receptor blockers caused greater hypotension at the night period in people with metabolic syndrome relative to exercise alone. However, other studies have refuted this hypothesis testing the same [52] or other medication [53] in hypertensive people, indicating the more evidence is needed. In addition, no studies with diuretics have been conducted.

We acknowledge some limitations of the present study. First, our findings are based on a small sample size. Second, obesity was not controlled as an eligibility criterion. Third, the possible mechanisms responsible for PEH were not assessed. Fourth, although exercise sessions were equalized by time, different caloric expenditures were likely provided by RES (124 kcal), AER (160 kcal), and COM (139.0 kcal) exercises [2]. Fifth, PEH was significant when postexercise was compared with preexercise values (PEH I), but not with the control session PEH II. Experts in the field have suggested that interpretations based on PEH II should be preferred over PEH I in studies using ABPM [54]. However, authors stated the importance of preexercise values to take into consideration day-to-day variation on BP [54]. Finally, the same baseline values were used for all comparisons.

Our findings have practical applications, given that low BP levels after acute exercise may contribute to low cardiovascular risk during the performance of ADL [23, 24]. Hence, health professionals responsible for exercise prescription in patients with RH should preferably prescribe COM exercise, instead of either AER or RES alone. In addition, acute hemodynamic responses to physical exercise may predict long-term adaptations [21, 55], suggesting that the chronic effects of COM should be tested in randomized clinical trials as tool for the management of blood pressure in people with RH.

## 5. Conclusion

Findings of the present study indicate that AER, RES, and COM exercises elicit systolic and diastolic postexercise ambulatory hypotension in RH patients. Notably, longer hypotension periods were observed after COM exercise. In addition, NON-RH and RH people showed different changes on BP after exercise sessions, suggesting that postexercise hypotension is influenced by the pathophysiological bases of hypertension.

## Figures and Tables

**Table 1:** General characteristics of resistant hypertensive (RH) subjects and non-resistant hypertensive (NON-RH) subjects according to clinical and biochemical data and antihypertensive (anti-HT) drugs used by the subgroups.

	RH	NON-RH	*P* value
	(*n* = 10)	(*n* = 10)	
Clinical data			
Age (years)	60 ± 9	54 ± 13	0.66
Female gender, *n* (%)	6 (60)	5 (50)	1
Diabetes mellitus, *n* (%)	10 (100)	5 (50)	0.09
BMI (kg/m^2^)	31 ± 5	32 ± 7	0.18
Fat-free mass (kg)	54 (43-71)	61 (47-82)	0.40
Fat mass (kg)	25 ± 10	26 ± 14	0.32
Total body water (L)	75 ± 2	75 ± 3	0.17
Basal metabolic rate (cal/day)	1765 ± 482	1996 ± 540	0.39
Biomarkers			
HbA1C (%)	7 ± 2	6 ± 0.7	0.13
Glucose (mg/mL)	97 (89-139)	98 (94-134)^∗^	0.07
Creatinine (mg/mL)	0.8 (0.1-1.1)	0.8 (0.7-0.9)	0.81
Aldosterone (pg/mL)	100 ± 141	132 ± 95	0.64
Creat clear (mL/min/1.73m^2^)	83 ± 66	89 ± 65	0.86
Cholesterol (mg/mL)	188 ± 48	175 ± 40	0.55
HDL-c (mg/mL)	44 ± 9	38 ± 7^∗^	0.07
LDL-c (mg/mL)	109 ± 35	106 ± 46	0.88
Triglycerides (mg/mL)	135 (90-214)	143 (105-205)	0.97
Anti-HT drugs			
Number of classes	4 ± 1	2 ± 1	0.13
Diuretics, *n* (%)	10 (100)	5 (50)^∗^	0.03
Spironolactone, *n* (%)	2 (20)	2 (20)	1
Beta-blockers, *n* (%)	8 (80)	6 (60)	0.63
ACEIs and ARBs, *n* (%)	5 (50)	7 (70)	0.65
CCBs, *n* (%)	8 (80)	3 (30)	0.07
Others, *n* (%)	0	0	1

Values are expressed as mean ± standard deviation or median (1st, 3rd quartiles), according to data distribution. RH: resistant hypertensive; NON-RH: non-resistant hypertensive; BMI: body mass index; HbA1C: glycated hemoglobin; Creat clear: creatinine clearance; LDL and HDL: low- and high-density lipoproteins, respectively; anti-HT: antihypertensive drugs; ACEIs: angiotensin-converting enzyme inhibitors; ARBs: angiotensin receptor blockers; CCBs: calcium channel blockers.

**Table 2:** Area under the curve of the experimental sessions.

	RES		AER		COM	
	Mean	SD	Mean	SD	Mean	SD
Resistant hypertension						
24 h						
SBP	2618	18.2	2610	15.2	2535ab	15.2
DBP	1049	11.5	1070a	11.7	1052b	9.8
Awake-time						
SBP	1871	18.5	1798	19.4	1778ab	16.3
DBP	787	12.0	771a	13.0	752ab	16.1
Night-time						
SBP	1356	17.2	1361a	18.2	1320ab	19.8
DBP	469	12.9	468	13.2	468	17.7
Nonresistant hypertension						
24 h						
SBP	1796	16.1	1767a	14.1	1823ab	16.5
DBP	1019	12.0	1004	13.3	1081ab	16.3
Awake-time						
SBP	1781	12.0	1741a	12.0	1816ab	15.6
DBP	1045	10.9	1005a	12.1	1090ab	17.1
Night-time						
SBP	954	17.3	947	15.6	976ab	19.8
DBP	365	11.4	373	12.8	397ab	17.7

SBP = systolic blood pressure; DBP = diastolic blood pressure; SD = standard deviation; ^a^*P* < 0.05 vs. strength; ^b^*P* < 0.05 vs. aerobic.

## Data Availability

Data are available upon reasonable request.
